# Hsa_circ_0081534 increases the proliferation and invasion of nasopharyngeal carcinoma cells through regulating the miR-508-5p/FN1 axis

**DOI:** 10.18632/aging.103963

**Published:** 2020-10-20

**Authors:** Sujuan Li, Qingshan Wang

**Affiliations:** 1 Department of Otology, The First Affiliated Hospital of Zhengzhou University, Zhengzhou 450052, Henan, China; 2 Department of Otorhinolaryngology, Weihai Central Hospital, Weihai 264400, Shandong, China

**Keywords:** nasopharyngeal carcinoma, hsa_circ_0081534, miR-508-5p, FN1

## Abstract

Accumulating lines of evidence indicate that circular RNAs (circRNAs) are involved in the pathogenesis of human cancers, including nasopharyngeal carcinoma (NPC). However, the influences of hsa_circ_0081534 upon the pathogenesis and dynamics of NPC are undescribed. In this study, we identified a circRNA hsa_circ_0081534 was significantly upregulated in NPC tissues and cell lines. Inhibition of hsa_circ_0081534 induced a decrease in NPC cells proliferation and invasion in vitro, and repressed tumor growth in vivo. In mechanism, hsa_circ_0081534 promoted NPC progression by sponging miR-508-5p. Fibronectin 1 (FN1) is a target gene of miR-508-5p. In addition, rescue assays showed that FN1 overexpression (or miR-508-5p inhibitors) abolished the roles of hsa_circ_0081534 inhibition on NPC cells proliferation and invasion. Therefore, hsa_circ_0081534 promoted the proliferation, and invasion of NPC cells via regulating the miR-508-5p/FN1 axis. Our findings suggested that hsa_circ_0081534 could be a novel therapeutic target for the treatment of NPC patients.

## INTRODUCTION

Nasopharyngeal carcinoma (NPC) is a common malignant head and neck cancer that affects humans on a global scale, and disproportionately affects a relatively high percentage of Chinese [1, 2]. Despite improvements in treatment approaches on therapeutic strategies such as radiotherapy and chemoradiotherapy, the prognosis and outcomes remain unsatisfying [3, 4]. The dynamics underlying the progression of NPC are complex, which is caused by the aberrant expression of oncogenes or anti-oncogenes [5]. Therefore, there is an urgent need to elucidate the mechanisms underlying the NPC progression to find better therapeutic strategies for the treatment of NPC.

Circular RNAs (circRNAs) are a new class of endogenous functional non-coding RNAs and characterized by covalently closed and continuous loop structures without 3’ or 5’ end [6]. In recent years, increasing evidence showed that circRNAs can function as sponges of microRNAs (miRNAs) [7]. The abnormal regulation of circRNAs was involved in the pathogenesis of human diseases, including cancers [8, 9]. For example, Shen et al. found that CircSERPINE2 overexpression alleviated human chondrocyte cell apoptosis as well as promoted anabolism of extracellular matrix by regulating the miR-1271/E26 pathway [10]. Garikipati et al. reported that circFndc3b modulated cardiac repair post-myocardial infarction via its influence upon the dynamics of FUS/VEGF-A axis [11]. Li et al. suggested that circPRRC2A promoted angiogenesis and metastasis through upregulation of TRPM3 by sponging miR-514a-5p and miR-6776-5p in renal cell carcinoma [12]. However, the specific mechanism of hsa_circ_0081534 on NPC development have not been fully elucidated, and require further investigation.

It is well documented that microRNAs (miRNAs) exert tumor-suppressive activity in different human malignancies [13]. Notably, circRNAs are known to interact with miRNAs to block miRNA-mediated target gene expression in NPC. For example, Rui et al. indicated that circSERPINA3 accelerated NPC cell growth by enhancing MDM2 via inducing inhibition of miR-944 [14]. Ke et al. found that CircHIPK3 bound with miR-4288 to induce expression of ELF3, thereby promoting NPC cell growth and metastasis [15].

Fibronectin 1 (FN1) is a member of the FN family, is widely expressed by multiple types of cells, and may facilitate the development of cancers [16, 17]. For example, Xun et al. found that inducing the inhibition of FN1 reduced colorectal carcinogenesis by suppressing proliferation and invasion [18]. Zhou et al. found that LINC00963 sponged miR-204-3p by targeting FN1 to promote osteosarcoma cells proliferation and invasion [19]. Moreover, Gao et al. found that miR-613 inhibited angiogenesis in NPC cells by triggering the inactivation of FN1-dependent AKT signaling pathway [20]. However, the potential regulatory mechanisms of hsa_circ_0081534 related to the miR-508-5p/FN1 axis in NPC is still unclear.

Therefore, in the present study, we sought to assess the influence of hsa_circ_0081534 in NPC progression. We also aimed to characterize crosstalk among hsa_circ_0081534, miR-508-5p, and FN1. Ultimately, we hoped our novel assessments and findings might lead to better treatment approaches and outcomes for patients afflicted with NPC.

## RESULTS

### hsa_circ_0081534 expression was upregulated in NPC

**Figure 1:**
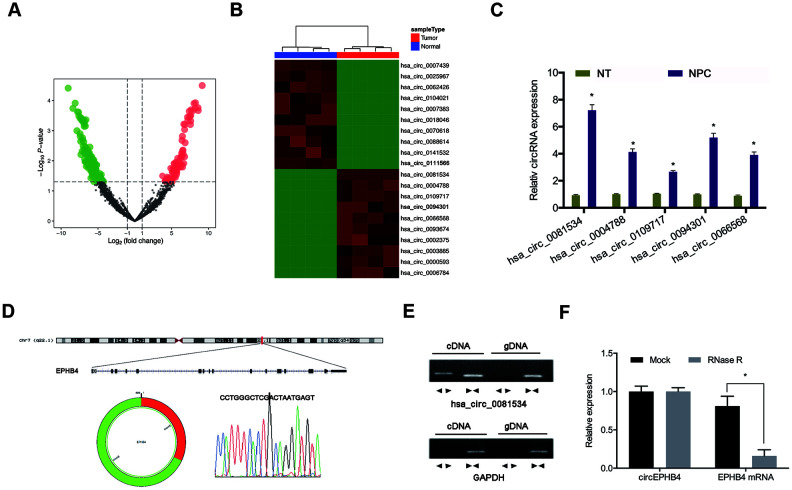
**Screening of NPC-related circRNAs in GSE143797.** (**A**, **B**) Hierarchical clustering analysis and volcano plots for significantly differentially expressed circRNAs in tumorous or adjacent normal tissues for NPC patients (N = 5). (**C**) Relative expression of 5 upregulated circRNAs in NPC tissues (n=8). (**D**) Schematic of hsa_circ_0081534. (**E**) hsa_circ_0081534 was assessed by use of divergent primer sets and amplified in cDNA but did not amplify by use of these primer sets in gDNA. (**F**) Levels of hsa_circ_0081534 were detected in NPC cells treated with RNase R, and for NPC cells not treated with RNase R. *P < 0.05.

To assess measures of involvement of circRNAs in the pathogenesis of NPC, we firstly analyzed microarray expression profiles (GSE143797) comparing circRNA levels between NPC tissues and normal tissues. Results indicated that expression of 296 circRNAs were significantly altered in NPC tissues compared to adjacent normal tissues (fold change > 2.0 and *p* < 0.05) (Figure1A, 1B). Next, we selected the top 5-upregulated circRNAs from dataset GSE143797 and sought to verify the predictions in small samples (N = 5) of NPC tissues by use of qRT-PCR. Results indicated that hsa_circ_0081534 (circEPHB4) was the most significantly upregulated type of circRNAs of those we assessed in the NPC tissues (Figure 1C).

Hsa_circ_0081534 (circEPHB4) was derived from exons 5 and 6 of the EPHB4 gene (Figure 1D). To rule out the possibility of genomic rearrangement or trans-splicing, we designed divergent primers to amplify hsa_circ_0081534. Results illustrated that hsa_circ_0081534 was amplified in cDNA, but was not amplified in gDNA (Figure 1E). RNase R assay revealed that hsa_circ_0081534 was resistant to RNase R (Figure 1F).

### Knockdown of hsa_circ_0081534 inhibited viability, and invasion in NPC cells

**Figure 2:**
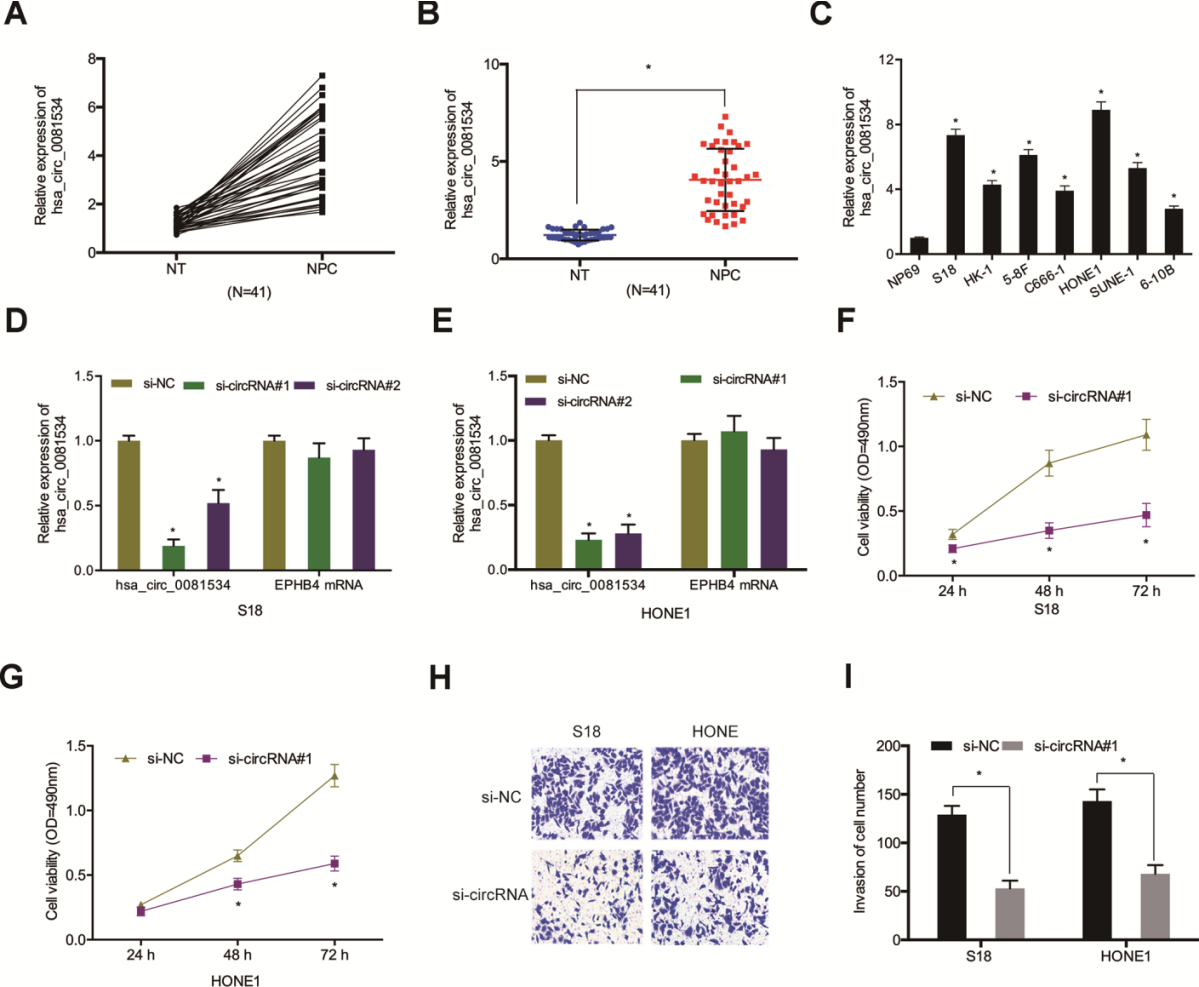
**Silencing of hsa_circ_0081534 induced a reduction in the progression of NPC cells in vitro.** (**A**–**C**) Relative expression of hsa_circ_0081534 in NPC tissues and cell lines. (**D**, **E**) qRT-PCR assessment of interference efficiencies of si-circ_0081534 in NPC cells. (**F**, **G**) Cell proliferation after silencing hsa_circ_0081534 was assessed by MTT assay. (**H**, **I**) Transwell assay was used to determine NPC cells invasion ability in vitro. NT: adjacent normal tissues; NPC: nasopharyngeal carcinoma tissues. *P < 0.05.

Next, we examined hsa_circ_0081534 expression in NPC tissues and cell lines. Results indicated that hsa_circ_0081534 expression increased in NPC tissues compared with adjacent normal tissues (NT) (Figure 2A, 2B). Similarly, hsa_circ_0081534 expression also was significantly enhanced in NPC cell lines (S18, HK-1, 5-8F, C666-1, HONE1, SUNE-1, and 6-10B) (Figure 2C). To investigate potential biological functions of hsa_circ_0081534, we transfected si-NC or si-circ_0081534 into S18 and HONE1 cells (Figure 2D, 2E). MTT assay revealed that si-circ_0081534 significantly decreased NPC cell proliferation in vitro (Figure 2F, 2G). Additionally, transwell assay indicated that inducing the down-regulation of hsa-circ_0081534 notably inhibited NPC cells invasion ability (Figure 2H, 2I). These data indicated that inducing the downregulation of hsa_circ_0081534 reduced NPC cells progression in vitro.

### Hsa_circ_0081534 bound to miR-508-5p and inhibited its expression

**Figure 3:**
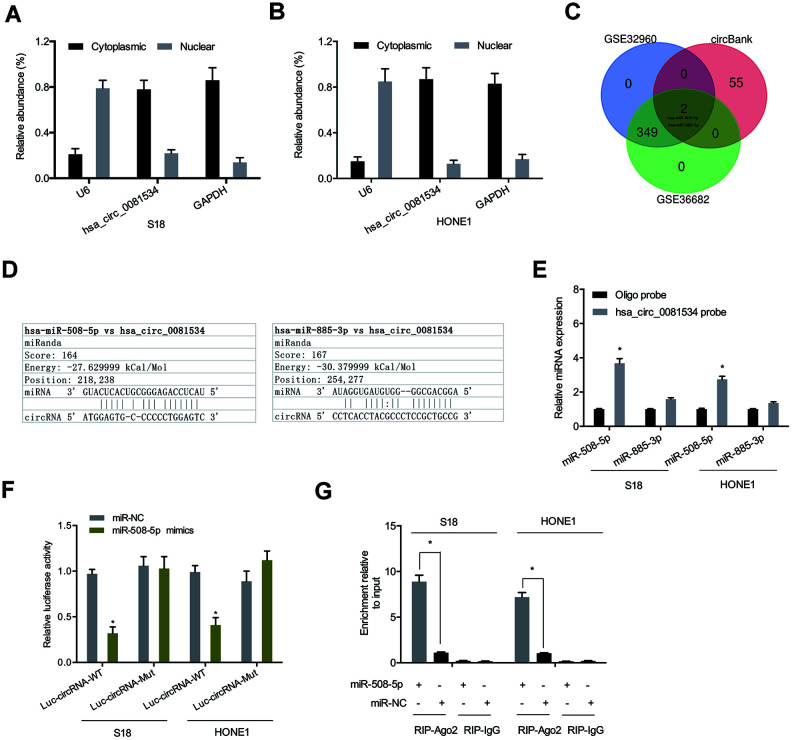
**Hsa_circ_0081534 bound to miR-508-5p.** (**A**, **B**) Subcellular fractionation assays were utilized to test for localization of hsa_circ_0081534. (**C**, **D**) A Venn diagram showing predicted binding sites between hsa_circ_0081534 and miR-508-5p. (**E**) Pull down assay showing that miR-508-5p was abundantly pulled-down by hsa_circ_0081534 probe. (**F**) Relative luciferase activity in NPC cells was measured by dual-luciferase reporter assays. (**G**) RIP assay facilitated the enrichment of hsa_circ_0081534 and miR-508-5p in NPC cells. *P < 0.05.

To assess mechanisms underlying the influence of hsa_circ_0081534 in NPC, we determined the location of hsa_circ_00815343 in NPC afflicted cells. Results indicated that subcellular localizations of hsa_circ_0081534 mainly occurred in cytoplasm (Figure 3A, 3B). Bioinformatic analyses suggested that both miR-508-5p and miR-885-3p were potential targets of hsa_circ_0081534 (Figure 3C, 3D). Pull down assay indicated that miR-508-5p was abundantly pulled-down by hsa_circ_0081534 probes in both S18 and HONE1 cells (Figure 3E). Next, correlations between hsa_circ_0081534 and miR-508-5p were further examined by use of dual-luciferase reporter and RIP assays (Figure 3F, 3G).

**Figure 4:**
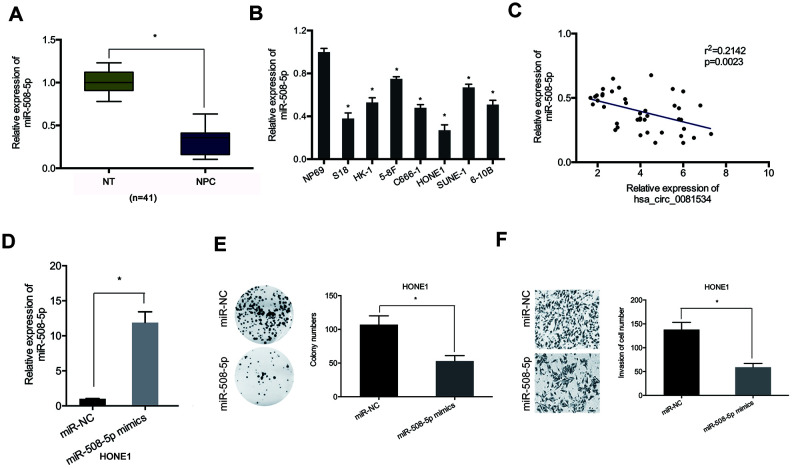
**miR-508-5p mimics decreased NCP cells proliferation and invasion.** (**A**, **B**) miR-508-5p expression in NPC tissues and cell lines. (**C**) MiR-508-5p expression was negatively correlated with hsa_circ_0081534 expression in NPC tissues. (**D**) Transfected efficiency of miR-508-5p mimics in HONE1 cells was determined by qRT-PCR. (**E**, **F**) miR-508-5p overexpression induced a subsequent reduction in HONE1 cells proliferation and invasion abilities. *P < 0.05.

We also detected miR-508-5p expression in NPC tissues and cells by using qRT-PCR. As shown in in Figure 4A, 4B, miR-508-5p expression in NPC tissues and cell lines was significantly reduced compared to adjacent normal tissues and cells. Correlation analysis showed that miR-508-5p expression associated inversely with hsa_circ_0081534 expression in NPC tissues (Figure 4C). Thereafter we examined the roles of miR-508-5p in NPC cells. Functional assays indicated that miR-508-5p overexpression significantly reduced proliferative and invasive abilities in NPC cells (Figure 4D–4F). Ultimately, these data suggested that hsa_circ_0081534 might function as a miR-508-5p sponge in NPC cells progression.

### FN1 was a target of miR-508-5p

**Figure 5:**
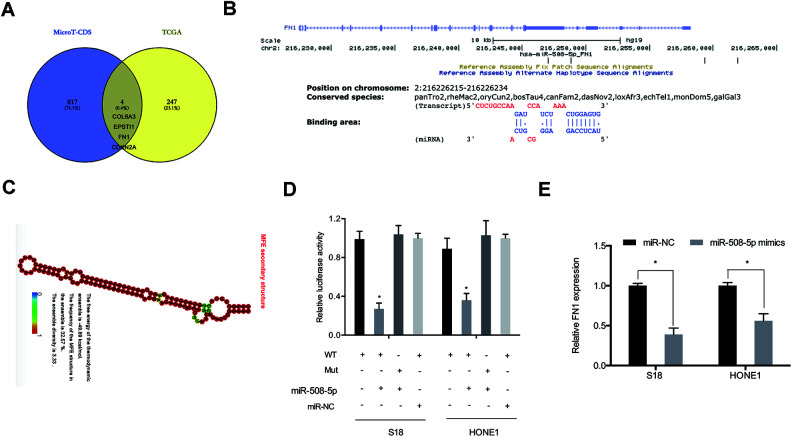
**FN1 was a target of miR-508-5p.** (**A**) Venn diagram indicating potential targets explored by MicroT-CDS and TCGA databases. (**B**) Predicted binding sites between miR-508-5p and FN1. (**C**) The secondary structure of miR-508-5p. (**D**) Examinations of combinations between miR-508-5p and FN1 were verified by dual-luciferase reporter assays in NPC cells. (**E**) FN1 expression was detected by qRT-PCR in NPC cells transfected with either miR-NC or miR-508-5p mimics. *P < 0.05.

We also sought to elucidate the mechanisms of miR-508-5p in the progression of NPC. Bioinformatics databases were utilized to determine potential targets of miR-508-5p. As shown in Figures 5A–5C, the 3’ UTR of FN1 had suitable binding sites with miR-508-5p. Dual-luciferase reporter assay indicated that overexpression of miR-508-5p notably decreased consequent measures of luciferase activity in the FN1-Wt group (Figure 5D). Next, qRT-PCR revealed that miR-508-5p mimics decreased FN1 expression levels in NPC cells (Figure 5E).

**Figure 6:**
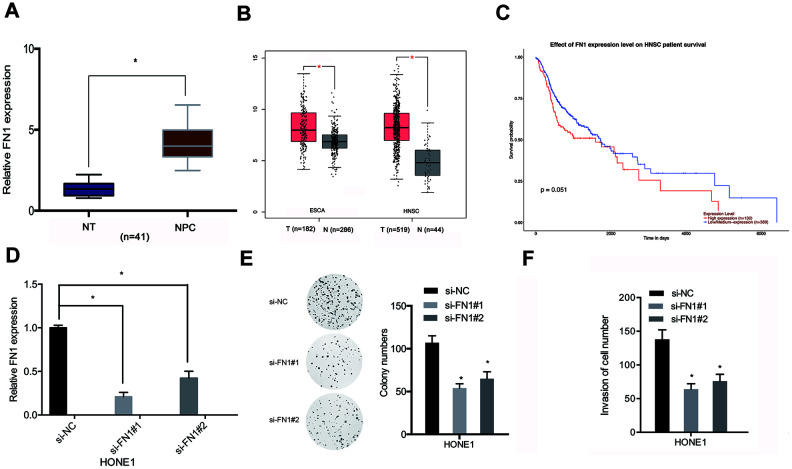
**FN1 knockdown inhibited proliferative and invasive abilities of NPC cells.** (**A**, **B**) FN1 expression levels in NPC tissues were analyzed through qRT-PCR and TCGA databases. (**C**) Relatively high levels of expression of FN1 were associated with poor prognosis for NPC patients. (**D**) The knockdown efficiency of si-FN1 in HONE1 cells. (**E**, **F**) Cell proliferative and invasive abilities post-knockdown of FN1 were determined through the use of colony formation and transwell assays. ESCA: esophageal carcinoma; HNSC: head and neck squamous cell carcinoma. *P < 0.05.

We next sought to assess FN1 expression in NPC samples. QRT-PCR results indicated that FN1 expression was relatively highly expressed in NPC tissues compared with adjacent normal tissues (Figure 6A). In addition, these findings were supported by the results from a search of the TCGA database (Figure 6B). Kaplan-Meier analyses indicated that elevated FN1 expression was significantly correlated with poorer prognosis for NPC patients (Figure 6C). Then, we examined the roles of FN1 in NPC cells. QRT-PCR indicated that FN1 was significantly downregulated in HONE1 cells (Figure 6D). Functional assays indicated that FN1 inhibition significantly reduced proliferative and invasive abilities of HONE1 cells (Figure 6E, 6F). These data supported that FN1 was a downstream gene of miR-508-5p in NPC cells.

### Hsa_circ_0081534 promoted NPC progression through the miR-508-5p/FN1 axis

**Figure 7:**
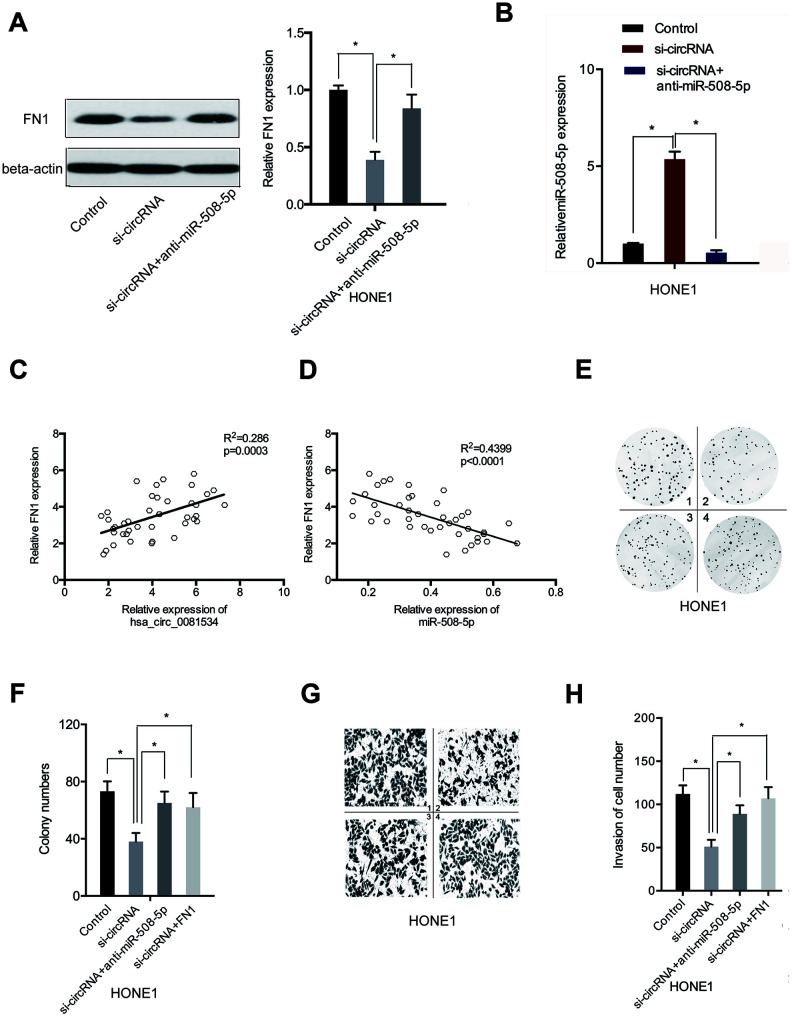
**The hsa_circ_0081534/miR-508-5p/FN1 axis in NPC.** (**A**) Levels of FN1 were examined in NPC cells transfected with si-NC, si-circ_0081534, or si-circ_0081534 + anti-miR-508-5p through the use of Western blot. (**B**) Levels of miR-508-5p were examined in NPC cells transfected with si-NC, si-circRNA, or si-circRNA + anti-miR-508-5p through the use of qRT-PCR. (**C**) FN1 expression positively correlated with hsa_circ_0081534. (**D**) FN1 expression negatively correlated with miR-508-5p. (**E**–**H**) FN1 overexpression (or miR-508-5p inhibition) abolished the roles of hsa_circ_0081534 knockdown on NPC cells proliferation and invasion. 1: control; 2: si-circRNA; 3: si-circRNA+ anti-miR-508-5p; 4: si-circRNA+ FN1. *P < 0.05.

Next, we explored if hsa_circ_0081534 influenced the progression of NPC through regulation of the miR-508-5p/FN1 axis. Findings from western blot indicated that silencing hsa_circ_0081534 subsequently decreased FN1 expression in NPC cells, and the effects could be reversed through inducing miR-508-5p inhibition (Figure 7A), and the expression of miR-508-5p was also explored (Figure 7B). Next, we assessed relationships among hsa_circ_0081534, miR-508-5p, and FN1. Association analyses indicated that hsa_circ_0081534 expression was positively correlated with FN1 expression in NPC tissues (Figure 7C). In addition, FN1 expression correlated inversely with miR-508-5p expression in NPC tissues (Figure 7D). Moreover, rescue assays suggested that FN1 overexpression (or miR-508-5p inhibition) subsequently reversed the results of hsa_circ_0081534 silencing on HONE1 cell proliferative (Figure 7E, 7F) and invasive abilities in vitro (Figure 7G, 7H).

### hsa_circ_0081534 reduced NPC growth in vivo

**Figure 8:**
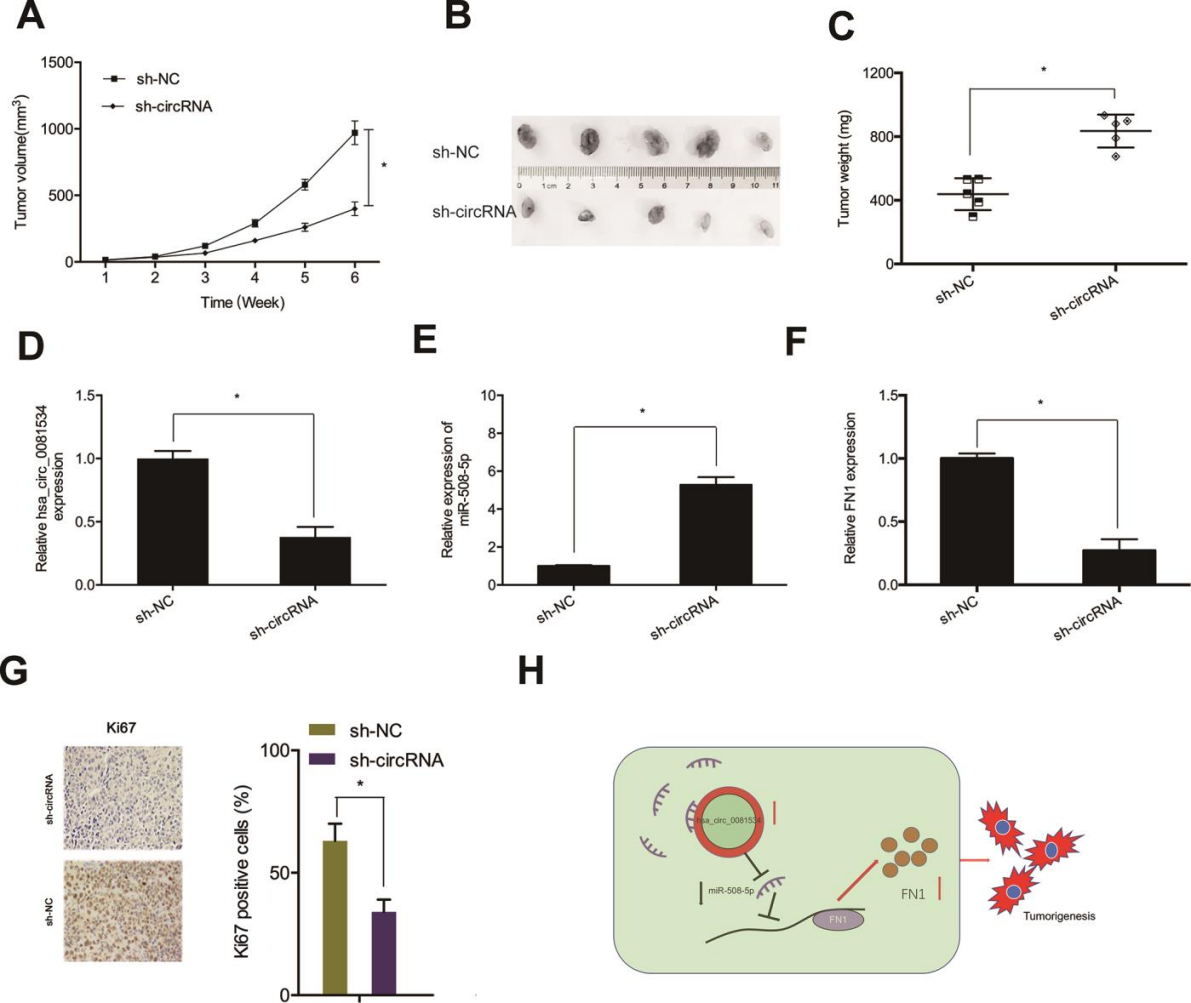
**Hsa_circ_0081534 knockdown blocked tumor growth in vivo.** (**A**) Tumor volume was measured every 7 days. (**B**, **C**) After 6 weeks, mice were sacrificed, and excised tumors were weighed. (**D**–**F**) Levels of hsa_circ_0081534, miR-508-5p, and FN1 were measured using qRT-PCR. (**G**) Hsa_circ_0081534 knockdown suppressed Ki-67 expression in mice. (**H**) Schematic diagram of the hsa_circ_0081534/miR-508-5p/FN1 axis in NPC progression. *P < 0.05.

To assess roles of hsa_circ_0081534 in vivo, we established a murine xenograft using S18 cells expressing sh-circ_0081534 or sh-NC. As shown in Figures 8A, hsa_circ_0081534 depletion subsequently inhibited NPC tumor growth in vivo. Furthermore, both dimensions and weights of tumors were smaller in the sh-circ_0081534 group compared with sh-NC group (Figure 8B, 8C). Abundances of hsa-circ_0081534, miR-508-5p, and FN1 were determined for NPC tissues dissected from mice. As shown in Figure 8D–8F, significantly decreased levels of expression of both hsa-circ_0081534 and FN1 were found in the sh-circ_0081534 group, whereas hsa-circ_0081534 depletion subsequently and notably increased miR-508-5p expression in NPC tumor afflicted tissues. In addition, Ki67 staining assay revealed that hsa_circ_0081534 knockdown subsequently suppressed tumor cell proliferation in vivo (Figure 8G). These outcomes indicated that hsa_circ_0081534 promoted NPC progression by sponging miR-508-5p to increase FN1 expression (Figure 8H).

## DISCUSSION

Recently, numerous of evidence has indicated that circRNAs play important roles in NPC pathogenesis. For example, Hong et al. showed that circular RNA CRIM1 functioned as a ceRNA to promote NPC metastasis and docetaxel chemoresistance through FOXQ1 regulation [21]. Li et al. showed that circ-ZNF609 depletion repressed proliferation of NPC cells via miR-188/ELF2 axis modulations [22]. Thus, herein, we examined the influence of the circRNA hsa_circ_0081534 in NPC progression. In the present study, hsa_circ_0081534 was highly expressed in NPC tissues and cell lines. Functional experiments revealed that hsa_circ_0081534 promoted the proliferative and invasive abilities of NPC cells in vitro. Furthermore, based upon our xenograft model, hsa_circ_0081534 depletion consequently impeded NPC tumor growth in vivo. Thus, these findings suggested the hypothesis that hsa_circ_0081534 could serve as an oncogenic circRNA with respect to the progression and pathogenesis of NPC.

Recently, accumulating evidence have indicated that circRNAs mediate tumorigenesis by acting as sponges, or by competing for endogenous RNAs (ceRNAs) of miRNAs [23]. In the current study, we used bioinformatics software, which helped us to predict 2 putative target miRNAs for hsa_circ_0081534. Consequently, miR-508-5p was selected for additional research. Independently derived conclusions have corroborated that miR-508-5p acted as an inhibitor in multiple types of cancers. For example, Liu et al. indicated that miR-508-5p acted as a prognostic marker, which suppressed both cell proliferation and migration in glioma [24]. Wu et al. showed that miR-508-5p acted as a tumor suppressor by targeting MESDC1 in liver cancer [25]. However, the roles and underlying mechanisms of miR-508-5p in NPC progression remain unclear. In the present study, hsa_circ_0081534 sponged miR-508-5p in NPC. Subsequently, overexpression of miR-508-5p reduced proliferative and invasive abilities of NPC cells in vitro. Moreover, rescue experiments revealed that inhibitory effects from inducing hsa_circ_0081534 knockdown on the malignancy of NPC cells could be reversed by miR-508-5p inhibitors. Therefore, si-circ_0081534 might attenuate tumorigenesis of NPC cells via increasing expression of miR-508-5p.

FN1 acts as direct transcriptional target of several miRNAs and involves in the tumorigenesis of multiply tumors, such as breast cancer, cervical cancer, and gastric cancer, et al. [26–28]. In this study, we found elevated FN1 expression in NPC tissues. High FN1 expression negatively correlated with miR-508-5p expression in NPC. Inducing the knockdown of FN1 expression subsequently reduced NPC cell proliferative and invasive abilities in vitro. Moreover, up-regulation of FN1 spurred the reversal of anti-tumor effects of hsa_circ_0081534 knockdown upon NPC cells progression. Therefore, we hypothesize that hsa_circ_0081534 might have exerted its tumor oncogenic effects through regulating the miR-508-5p/FN1 axis in NPC.

## CONCLUSIONS

In summary, these findings demonstrated that hsa_circ_0081534 potentiated the proliferative and invasive capabilities of NPC cells through up-regulating FN1 via sponging of miR-508-5p. Therefore, our findings provided a novel therapeutic target for the treatment of NPC patients.

## MATERIALS AND METHODS

### Clinical samples and cell culture

Patients diagnosed with NPC (N = 41) were recruited from The First Affiliated Hospital of Zhengzhou University. NPC tissues and adjacent normal tissues (NT) were collected during surgery. Prior to surgery, patients had not received any treatments. I Informed consent form was acquired from every patient. All procedures in our study were reviewed approved by the Ethics Committee of our hospital.

7 NPC cell lines (S18, HK-1, 5-8F, C666-1, HONE1, SUNE-1, 6-10B) and a human bronchial epithelial cell line (NP69) were bought from the Chinese Academy of Science (Shanghai, China). Cell lines were cultured in Dulbecco’s Modified Eagle’s Medium (DMEM, Invitrogen, Carlsbad, CA, USA) and supplemented with 10 % fetal bovine serum (FBS, Invitrogen) at a constant temperature of 37 °C and in an atmosphere containing a constant level of 5 % CO_2_.

### Transfection

Small interfering RNA against hsa_circ_0081534 (si-circ_0081534), miR-508-5p mimics, and miR-508-5p inhibitors and respectively matched controls were obtained from RiboBio (Guangzhou, China). The overexpression vector for FN1 and the negative control (pcDNA) were bought from GenePharma (Shanghai, China). Cell transfection procedures were completed by the application of Lipofectamine 3000 (Invitrogen) following all manufacturer protocols.

### RNase R assay

In RNase R assay, we used 2 μg of RNA, which was incubated either with, or without RNase R (3 U/μg; Epicentre, Madison, WI, USA) for 30 min at 37 °C. After the treatment above, RNA was transcribed into cDNA, and the expression was determined by qRT-PCR assay.

### RNA isolation and quantitative real-time PCR (qRT-PCR)

RNA was extracted from tissues and cells using TRIzol reagent (Invitrogen, Carlsbad, CA, USA). Following all manufacturer protocols, reverse transcription of miR-508-5p was conducted using the TaqMan MicroRNA Reverse Transcription Kit (Applied Biosystems, Foster City, CA, USA). The cDNA of hsa_circ_0081534 and of FN1 was synthesized using the PrimeScript Reverse Transcriptase Kit (Takara, Osaka, Japan). Amplification reactions were conducted using SYBR-Green Master Mix (Takara). Expression levels were analyzed using the 2^−ΔΔCt^ method [29]. U6 and GAPDH were used as controls.

### Western blotting

Total protein from tissue samples was extracted using lysis buffer (Beyotime Biotechnology). Next, we subjected proteins to 10 % SDS-PAGE, and then, proteins were electro-transferred onto a PVDF membrane (Millipore). Subsequently, membranes were blocked using 5 % non-fat milk, incubated with primary antibodies, and then were incubated with corresponding secondary antibodies. Finally, signals corresponding to levels of protein in samples were determined using the ECL Detection System (Thermo Fisher Scientific, Waltham, MA, USA). Blots were analyzed using ImageJ software (NIH, Bethesda, MD, USA) [30]

### RNA isolation from nuclear and cytoplasmic fractions

Both nuclear and cytoplasmic fractions for NPC cells were isolated using the PARIS kit (Invitrogen) and following methods detailed in a previous study [31].

### MTT assay

Transfected NPC cells were plated in 96-well plates and incubated for 24, 48, or 72 h. Next, cells were mixed with 20 μL of MTT (5 mg/mL; Invitrogen) for a further 4 h period of incubation. Formazan products were dissolved using 150 μL of DMSO. We recorded absorbance values at 490 nm and detections were facilitated by use of a microplate reader (Bio-Rad, Hercules, CA, USA).

### Colony formation assay

Transfected NPC cells were suspended and inoculated into 6-well plates (200 cells/well). After 14 days, cells were immobilized by using 4 % paraformaldehyde and were dyed with 0.1 % crystal violet. The colonies were photographed by a camera and counted using a microscope (Nikon, Japan).

### Transwell invasion assay

Transfected NPC cells were inoculated into the upper chamber of transwell chamber (Corning, NY, USA), which was covered by 50 μL of Matrigel. Cells were maintained in serum-free medium. The complete medium with added 10 % FBS was mixed into the lower transwell chamber. After 24 h of cultivation, cells were immobilized by use of 4 % paraformaldehyde and dyed with 0.1 % crystal violet. Measures of invasion of cells were determined under microscopy (Nikon, Japan).

### Dual-luciferase reporter assay

According to bioinformatics tool derived predictions, miR-508-5p was expected to have interacted with hsa_circ_0081534 or FN1 3’ UTR. To confirm this interaction, wild- and mutant-types of hsa_circ_0081534 or FN1 3’ UTR (WT/MUT-circ_0081534 or WT/MUT-FN13’UTR) were cloned into the pGL3 vector (Promega). Next, every constructed sample and miR-508-5p or miR-NC were co-transfected into NPC cells using Lipofectamine 3000. 24 h post-transfection, luciferase activities were evaluated by use of dual-luciferase assay kits (AmyJet Scientific, Wuhan, China) following all manufacturer protocols.

### RIP assay

Measures of combinations between miR-508-5p and hsa_circ_0081534 were assessed using Magna RIP Assay Kits (Millipore) following methods detailed in previous research [32].

### RNA-pull down assay

Biotin-labeled miR-NC or miR-508-5p were respectively named as Bio-miR-NC and Bio-miR-508-5p. Biotin-coupled complexes were immunoprecipitated, and qRT-PCR facilitated determinations of levels of enrichment of miRNA.

### Tumor formation in mice

A total of N = 10 male BALB/c nude mice (4 weeks old) were obtained from Beijing HFK Bioscience Co., Ltd (China, Beijing) and used for conducting xenograft assays. Briefly, stably expressing sh-NC, sh-circ_0081534 (sh-circRNA) [33] transfected S18 cells (1 × 10^6^ cells) were injected subcutaneously into mice. Tumor volume (length × width^2^ × 0.5) was assessed and recorded every seven days through 6 weeks post-injection. At 6-weeks post-injection, mice were anesthetized and sacrificed by cervical dislocation whereafter tumors were immediately removed and weighed. Animal experiments were conducted following the National Animal Care and Ethics Institution guidelines and were authorized by the Animal Research Committee of The First Affiliated Hospital of Zhengzhou University.
